# Supplementary material for: High Throughput Sequencing-Aided Survey Reveals Widespread Mixed Infections of Whitefly-Transmitted Viruses in Cucurbits in Georgia, USA
Source: Viruses. 2021 May 26;13(6):988. doi: 10.3390/v13060988 (PMC8230054; doi:10.3390/v13060988)
Supplement: Supplementary file 1 [file viruses-13-00988-s001.zip › viruses-1148822-si.pdf]

Supplementary Figure S1: Cucurbit growing counties in Georgia (light blue). Samples were collected from Colquitt, Worth and Tift (dark blue) representing the major cucurbit growing counties.

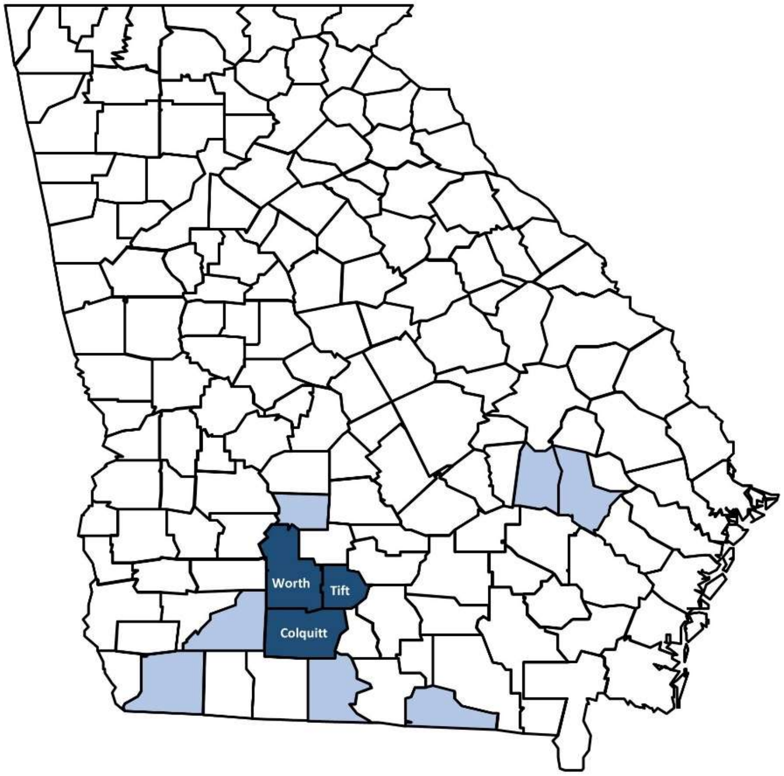

Supplementary Figure S2: Phylogenetic relationship of partial coat protein gene sequences of cucurbit yellow stunting disorder virus (CYSDV) isolates from Georgia (Assembled from siRNA sequences) with the corresponding sequences of CYSDV isolates available in the GenBank. The country of origin of the sequences used are shown in parentheses after the accession numbers of viruses. Construction of the phylogenetic trees were done by the neighbor-joining method. The number on each node shows the percentage of bootstrap values (2000 replicates) in which a given node was recovered. Nodes with lower than 50 bootstrap value were collapsed since they were insignificant.

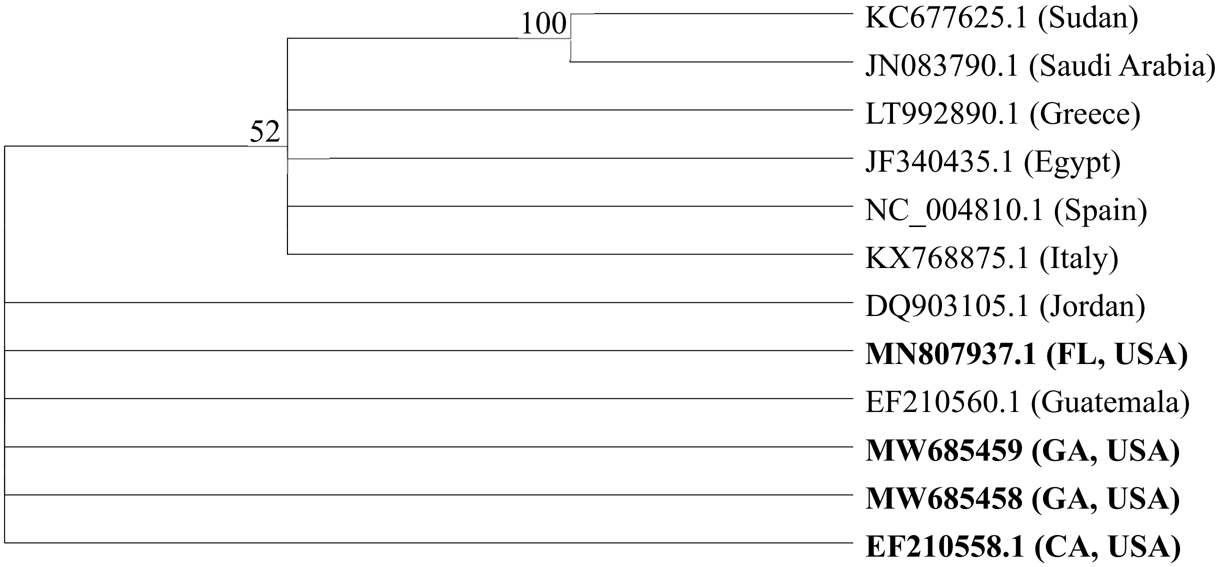

Supplementary Table S1. Read statistics of small RNA libraries prepared from symptomatic cucurbit samples.

Crop	Sample Names*	Total number of raw reads	No of reads after trimming	Average length after trimming
**Cantaloupe**	CaF1-2019	12,160,783	10,618,741	24
	CaF1-2020	22,257,504	21,820,714	21
	CaF2-2019	12,546,697	19,719,105	22
**Cucumber**	CF1-2019	11,725,746	11,217,872	23
	CF2-2019	14,177,959	12,342,530	22
	CF1-2020	22,297,989	21,914,383	23
**Squash**	SF1-2019	21,627,355	21,314,433	22
	SF1-2020	17,428,537	17,153,993	22
	SF2-2020	22,143,767	21,666,240	22

*In the sample names, 'Ca,' 'C' and 'S' denote the crop, F1 and F2 represent the field, 2019 and 2020 is the year in which the samples were collected.

Supplementary Table S2. Distribution of viruses in different counties in Georgia in 2019 and 2020.

Crop Virus	Colquitt County							Tift county			Worth County		
	Cantaloupe		Cucumber		Squash		Zucchini	Cucumber	Squash		Cucumber	Squash	Zucchini
	**2019**	**2020**	**2019**	**2020**	**2019**	**2020**	**2020**	**2020**	**2019**	**2020**	**2020**	**2020**	**2020**
CuLCrV	6	21	36	34	88	50	39	13	107	207	19	17	13
CySDV	12	36	6	24	27	43	5	17	4	99	37	19	20
CCYV	21	20	53	12	36	26	6	20	2	217	39	20	17
CuLCrV+ CySDV	4	14	2	22	24	43	5	10	3	95	17	16	13
CuLCrV+ CCYV	6	10	31	12	39	26	6	13	2	177	18	16	13
CySDV +CCYV	12	18	6	5	9	23	1	17	0	86	36	19	17
CuLCrV+ CySDV + CCYV	4	9	2	5	9	23	1	10	0	83	17	16	12
Total number of samples	**21**	**40**	**68**	**36**	**100**	**50**	**40**	**20**	**113**	**252**	**40**	**20**	**20**

Note. All samples collected in 2020 were also tested for cucurbit aphid borne yellows virus (CABYV), squash vein yellowing virus (SqVYV), and sida golden mosaic virus (SiGMV). These viruses were not detected in any of the samples tested.
